# Development, field-testing and optimization of tools to quantify soil-transmitted helminths in fecal sludge from school pit latrines in Ethiopia

**DOI:** 10.1371/journal.pntd.0014724

**Published:** 2026-09-15

**Authors:** Abebaw Tiruneh, Zeleke Mekonnen, Sara Roose, Mio Ayana, Fiona Vande Velde, Emmanuel C. Mrimi, John S. Gilleard, Michael R. Templeton, Zewdie Birhanu, Jaco J. Verweij, Luc E. Coffeng, Bruno Levecke

**Affiliations:** 1 School of Medical Laboratory Sciences, Institute of Health, Jimma University, Jimma, Ethiopia; 2 Department of Translational Physiology, Infectiology and Public Health, Ghent University, Merelbeke-Melle, Belgium; 3 Environmental Health and Ecological Science Department, Ifakara Health Institute, Dar es Salaam, Tanzania; 4 Faculty of Veterinary Medicine, University of Calgary, Alberta, Canada; 5 Department of Civil and Environmental Engineering, Imperial College London, London, United Kingdom; 6 Department of Health, Behavior, and Society, Institute of Health, Jimma University, Jimma, Ethiopia; 7 Microvida Laboratory for Medical Microbiology and Immunology, Elisabeth Tweesteden Hospital, Tilburg, The Netherlands; 8 Department of Public Health, Erasmus MC, University Medical Center Rotterdam, Rotterdam, The Netherlands; University of Melbourne, AUSTRALIA

## Abstract

**Background:**

Surveys to monitor large-scale deworming programs against soil-transmitted helminthiases (STH) involve collection and examination of stool samples from schoolchildren. These surveys are resource demanding and interrupt school activities. A potentially cost-saving alternative that does not involve children is to process fecal sludge samples from school pit latrines. As a first step towards a proof-of-principle of latrine-based monitoring of STH programs, we optimized tools to collect fecal sludge and to quantify STH eggs in the samples.

**Methods:**

First, we developed and field-tested three locally made fecal sludge sampling prototypes. Second, we developed a modified egg counting method and conducted spiking experiments to explore its analytical performance. Third, we estimated the variation in egg counts in fecal sludge samples that were collected from six primary schools in Ethiopia at different pit latrine depths used by boys and girls and that were repeatedly examined. Finally, field data was used to inform an egg count simulation model to quantify the variation in egg counts and to determine the sampling and analysis strategies that resulted in surveys as precise as stool-based surveys.

**Results:**

The modified fecal sludge sampling prototypes were generally successful, except for a few pit latrines with dried/solid fecal sludge and insufficient sludge volume. The egg counting method had moderately high analytical sensitivity that varied across the consistency of the samples and examination effort. The variation in egg counts was mainly explained by differences between squat holes followed by repeated fecal sludge sample processing. Latrine-based surveys were as precise as stool-based surveys only for *Ascaris* and when the intensity of infections was low.

**Conclusions:**

We developed a sampling and diagnostic strategy that we will use in a follow-up study which will be conducted in Jimma Zone across 25 schools (52 children per school) and will compare the mean fecal sludge egg counts at school level with the STH prevalence in children.

## Author summary

Progress of large-scale deworming programs are currently monitored through screening individual stool samples of schoolchildren. A latrine-based survey, without active participation of schoolchildren and interruption of routine school activities, is a potentially cost-effective alternative. As a proof-of-concept, we developed a method to quantify worm eggs in fecal sludge samples, conducted spiking experiments to determine its analytical performance and applied the method across schools in Ethiopia to explore variation in egg counts (e.g., squat holes, depth of sample collection, and repeated analyses). Based on our findings, we then determined the sample collection and laboratory analysis strategy that results in a latrine-based survey as precise as a survey based on screening individual stool samples. The analytical performance was moderately high but varied across the consistency of the samples and diagnostic efforts. The variation in egg counts was mainly driven by variation between squat holes on the same pit latrine, and thus it is better to sample more squat holes than repeatedly processing the same fecal sludge sample. We will now apply this sample collection and analysis across 25 schools (52 children per school) in Ethiopia and compare it with a survey based on screening individual stool samples.

## Introduction

Soil-transmitted helminthiases (STH) are caused by *Ascaris lumbricoides*, *Trichuris trichiura* and hookworms (*Necator americanus* and *Ancylostoma duodenale*). These diseases are transmitted through a sequence of events including (i) infected individuals excreting eggs laid by female adult worms into the environment through stool; (ii) the excreted eggs developing into infectious life stages; (iii) the life stages passively (oral uptake) or actively (skin penetration) entering individuals, and (iv) maturing to adult worms in the intestines. Because of this route of transmission, the diseases persist in settings where open defecation is frequently practiced, and where there is lack of access to clean water, basic sanitation, and low hygiene standards [1–3]. STH-attributable morbidities are mainly associated with moderate-to-heavy intensity (MHI) infections, resulting in malnutrition, physical and cognitive retardation in children, and anemia and negative birth outcomes (e.g., low birth weight) in women of reproductive age [2,4–6]. To reduce these morbidities, the World Health Organization (WHO) recommends large-scale deworming programs in endemic areas, during which anthelmintic drugs are periodically administered to at-risk populations [7,8], including but not limited to children.

Between 2010 and 2019, a significant reduction in the disease burden was observed (2.7 million DALYs in 2010 *vs.* 1.9 million DALYs in 2019) [9]. Encouraged by these global successes, WHO has now moved away from program coverage targets, and has defined new targets for 2030 that better reflect the maturity of the STH programs [10]. The targets include sustaining elimination of STH as a public health problem in endemic countries (prevalence of MHI infection in children <2% (**target #1**)) and a 50% reduction of tablets of anthelmintic drugs needed in STH control programs (**target #2**). As both targets are dependent on the outcome of epidemiological surveys to verify an elimination status (target #1) or to guide decisions on scaling down or stopping the administration of tablets (target #2), close monitoring of these programs is of utmost importance [11].

Today, the recommended monitoring and evaluation (M&E) tool is based on screening of stool samples from 250 schoolchildren across five schools (50 children per school) within a district using Kato-Katz thick smear technique [2,10,11]. However, recent studies questioned whether this survey design allows for reliable program decision-making, and screening of individual stool samples remains resource intensive [12–15]. A substantial proportion of the cost of performing these surveys is related to the individual stool sampling (transportation and per diem), followed by the process to prepare and count eggs in a stool smear [16,17]. As such, major cost drivers of M&E surveys are the number of schools and children to be screened, the speed at which technicians can process a single sample, the number of samples that can be processed per day, and thus the number of sampling days [18,19]. Although pooling samples [17,20] has been considered a cost-saving strategy, this proved to be sobering. Indeed, a pooling strategy mainly reduces the laboratory time, and not the resources required to collect the individual stool samples [17]. Therefore, it is important to explore alternative surveillance tools to monitor STH control programs [21].

A potential cost-saving strategy that merits more research is monitoring the environmental contamination (e.g., soil, wastewater, and fecal sludge) rather than the infections in children. This could allow screening a larger sample of the population at the same operational cost, and ultimately in more reliable program decision-making [22–27]. Monitoring the environmental samples also takes away important operational obstacles that programs are currently facing (e.g., expedites ethical process for stool samples and avoids class interruptions during surveys). Yet to date, there is little to no evidence that monitoring the environment is a cost-efficient alternative to inform STH control programs [6].

In our previous work, we demonstrated that soil samples from school compounds (playground, areas around the latrines and in the classrooms), households and open markets were highly contaminated with worm life stages, including but not limited to those causing STH. The environmental contamination at school level was associated with the prevalence of any STH across random sample of the children at those schools [25]. Here too, we encountered some important operational challenges related to both sampling and diagnostic strategy. While our samples were easy to collect (a shovel was the main equipment), it is not clear what number of samples should be collected to allow for an accurate and precise assessment of the environmental contamination. Our diagnostic method had a moderate detection limit (50 eggs per 100 g of soil for *Ascaris* and *Trichuris* eggs using microscopy), but it required expensive equipment (price: 8,000 EUR) to purify the eggs from the soil. Based on these challenges, we moved away from sampling soil and want to explore the potential of examination of fecal sludge samples from pit latrines instead.

As a first step to provide a proof-of-principle of a latrine-based M&E of STH control programs, this study aimed (i) to develop low-cost devices to collect fecal sludge samples, (ii) to develop a method to quantify STHs in fecal sludge samples under laboratory conditions, (iii) to field-test both the sample collection devices and the egg-counting method, and (iv) to determine the sampling (the required number of fecal sludge samples) and the analytical (number of preparations per sample and number egg counts per sample preparation) strategies to reliably assess the density of STH eggs in school pit latrines in Ethiopia.

## Methods

### Ethics statement

Ethical approval was obtained from the National Research Ethics Review Committee under The Ministry Education (Ref. No: 8/143/732/25) based on a support letter from Institutional Review Board of Jimma University, Ethiopia (Ref. No: JUIH/IRB/287/24). Similarly, ethical approval was received from Ghent University, Belgium (Ref. No: ONZ-2024-0364). Letters from Molecular Biology and NTD Research Center were distributed to each primary school involved in the study, and permission was sought from the schools’ administrations to collect fecal sludge samples from school pit latrines. We also obtained verbal informed consent from household heads to collect fecal sludge samples from three household pit latrines for spiking experiment.

### Development of low-cost fecal sludge sampling devices

We performed an exploratory review of scientific literature and targeted Google searches to identify existing devices for *in situ* grab sampling of fecal sludge from pit latrines at different depths. Guided by two key criteria - (i) low production cost and (ii) feasibility of local manufacture using readily available materials - we assessed whether a suitable, readily available sampler could be used or whether a new device needed to be designed. Our review was non-systematic and intended to inform the selection or development of appropriate fecal sludge sampling devices rather than to provide a comprehensive overview. Based on the insights gained, we designed prototype sampling devices. The suitability of the prototypes was subsequently evaluated under field conditions in seven primary schools (six schools in Jimma Town and one school in Manna District, Southwest Ethiopia).

### Quantification of STH eggs from fecal sludge samples

To quantify STH eggs in fecal sludge samples, we adapted our previously developed and validated diagnostic method for soil samples [25]. Briefly, the method for soil samples was based on (i) mixing samples in a liquid phase containing detergents (tween 80), (ii) sieving samples over a tower of sieves that were automatically shaken and washed, and further concentrating the eggs through (iii) steps of centrifugation, filtration and flotation.

For the fecal sludge samples, we simplified the procedure by replacing the automated sieving and washing machine with the ‘Fluke Catcher’ and using only water instead of detergent solutions.

**Fig 1:**
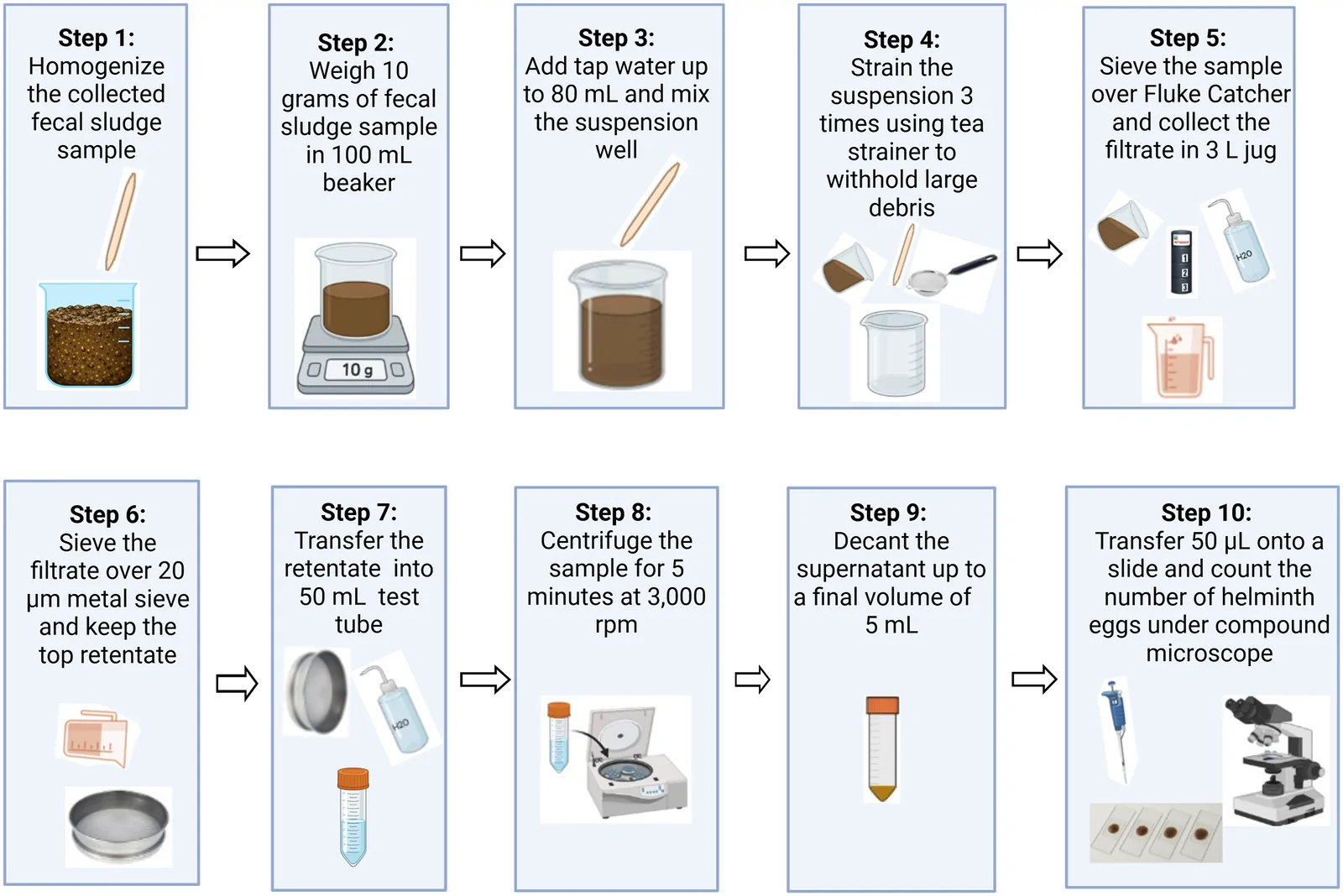
An overview of the procedure to quantify STH eggs in fecal sludge samples. The procedure consists of 10 steps including: homogenizing the collected fecal sludge sample in the original container (fecal sludge sample bucket) to uniformly distribute of eggs in the fecal sludge **(Step 1)**, weighing 10 g of fecal sludge sample using digital balance in 100 mL beaker **(Step 2)**, adding tap water until 80 mL and mixing the suspension **(Step 3),** straining the sample 3 times using tea strainer to withhold the large debris **(Step 4)**, straining the sample over Fluke Catcher and collecting the filtrate in a 3 L jug **(Step 5)**, straining the filtrate over 20 µm metal sieve **(Step 6)**, transfer the retentate into a 50 mL test tube including the rinsing water of the metal sieve **(Step 7)**, centrifuging the test tube for 5 min at 3,000 rpm **(Step 8)**, decanting the supernatant up to a final volume of 5 mL **(Step 9)** and transferring 50 µL onto a slide and counting the STH eggs using a compound microscope (10x10 magnification) **(Step 10)**. This figure was created using BioRender.com (https://www.biorender.com).

The Fluke Catcher is a hand-held stack of three sieves (sieve 1: 189 μm, sieve 2: 104 μm and sieve 3: 59 μm; all with diameter of 6 cm, and a total height of 32 cm). It is made commercially available by Provinos (price: 109.50 EUR) [28] to detect *Fasciola* eggs in feces of ruminants. Because STH eggs pass through all sieves of the Fluke Catcher, we decided to use an additional metal sieve of 10 cm (diameter) x 4 cm (height) and a mesh size of 20 μm (price: 369 EUR) [29]. Fig 1 briefly describes the procedures, and a detailed standard operating procedure (SOP) is available in S1 Text.

### Spiking experiments

For the spiking experiments, we collected two fecal sludge samples from pit latrines of three households and screened them using our modified Fluke Catcher method. To ensure that these fecal sludge samples were negative for helminth eggs, each sample was processed four times (each time processing a subsample of 10 g of the original fecal sludge) and three slides (each representing a volume of 50 µL) were examined for each sample preparation. From here onwards, we will refer to ‘sample preparation’ as a subsample of 10 g of the original fecal sludge that was processed using the developed method and ‘slide’ as a 50 µL of the sample preparation that was transferred on a microscope slide for microscopic examination.

To mimic the three types of fecal sludge consistencies, we measured the total solids (TS) of the collected two fecal sludge samples and diluted them (when needed) with tap water to obtain semi-solid (TS: 15.1-25%), slurry (TS: 5.0-15.0%) and liquid (TS: < 5%) samples [30]. The *Ascaris* eggs were obtained from both *Ascaris*-positive stool and fecal sludge samples using our soil-straining flotation method and stored in a stock volume of 3 – 5 mL at 2–8 ^°^C. We then examined the 50 µL of the stock solution in triplicate to estimate the concentration (number of purified *Ascaris* eggs per 1 µL).

To evaluate the analytical performance of our modified Fluke Catcher method, we spiked 50, 100, 200 and 400 purified *Ascaris* eggs into negative 10 g fecal sludge samples of different types; semi-solid, slurry and liquid. The spiking was repeated eight times for each combination of number of spiked *Ascaris* eggs and fecal sludge type, resulting in 96 spiked samples (8 replicates x 4 number of spiked eggs x 3 types of fecal sludge samples) and 384 worm egg counts (96 spiked samples x 4 slides of 50 µL). All samples were then processed following the SOP for the modified Fluke Catcher to quantify STH eggs in fecal sludge sample as described above. To avoid any systematic error, the spiking experiment was randomized for number of *Ascaris* eggs spiked and type of fecal sludge. The laboratory staff who performed the sample processing and microscopy examinations were blinded.

### Field evaluation of the fecal sludge sampling devices and the modified Fluke Catcher method

#### School selection.

We collected fecal sludge samples from six primary schools in Jimma Town, Southwest Ethiopia. The schools were selected based on their involvement in previous epidemiological, drug efficacy trial and diagnostic performance assessment surveys [20,25,31,32]. For each school, we first identified and inspected all pit latrines available in the school compound. When sampling fecal sludge was not possible from at least one pit latrine, for example when the fecal sludge had dried out, we proceed to collect samples from the next school.

#### Fecal sludge sampling and processing.

**Fig 2:**
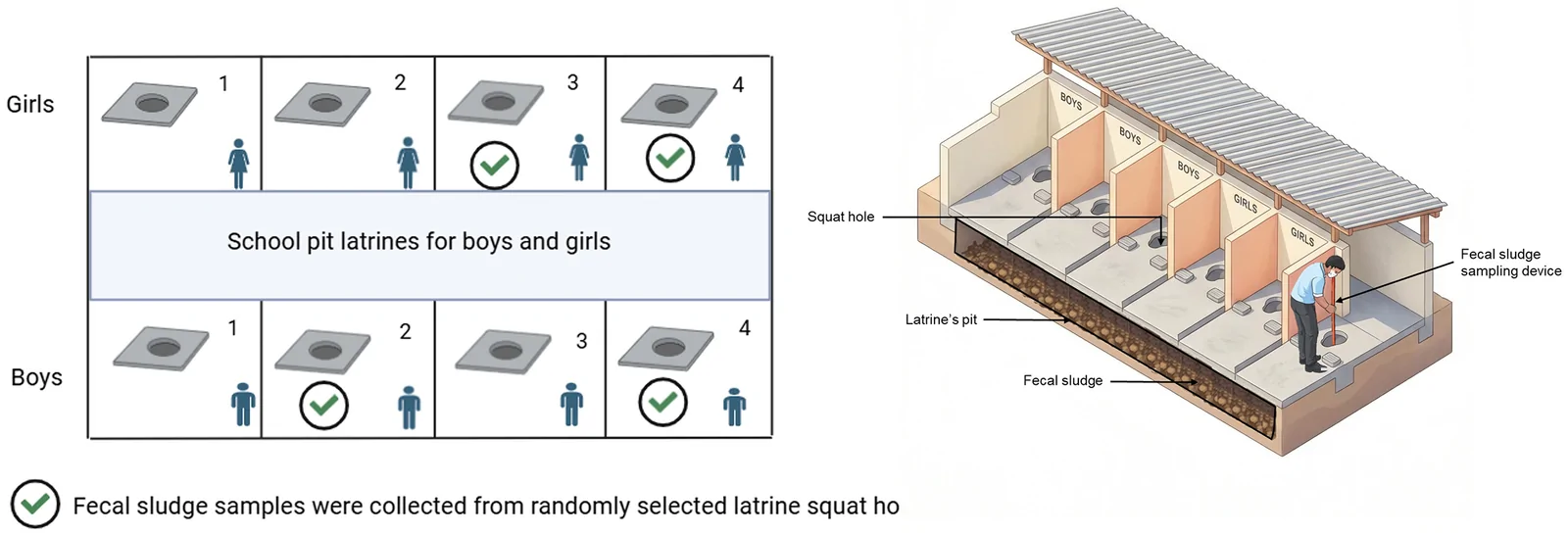
The selection of squat holes for the collection of fecal sludge samples. All pit latrines in each school compound were identified, and their eligibility was checked (eligible implied that pit latrines were used and fecal sludge sample collection was feasible). Eligible pit latrines were sketched on paper and squat holes were assigned a number (1 to the total number of squat holes). A squat hole was defined as an individual defecation opening on a pit latrine. Multiple squat holes located on the same pit latrine were considered separate squat holes. Two squat holes for each sex (boys and girls) were randomly selected (e.g., indicated with a tick mark on the paper sketch (left) and a fecal sludge sampling device inserted in squat holes (right)), and fecal sludge samples were collected at different depths (superficial and deep) from all randomly selected squat holes (right). The image was generated using OpenAI’s ChatGPT image-generation tools (OpenAI). The generated image was reviewed and approved by the authors. OpenAI Terms of Use are available at the OpenAI Terms of Use webpage (https://openai.com/policies/terms-of-use/).

Fecal sludge samples were collected from individual defecation openings on a pit latrine (squat holes). We did consider multiple squat holes located on the same pit latrine as separate squat holes. For the school pit latrines that were shared by both boys and girls, we collected fecal sludge samples from two latrine squat holes per sex (the sample size was pragmatically chosen to generate preliminary insights into the sources of variation in egg counts and to inform the simulation study) (Fig 2). When pit latrines were separated for boys and girls, we randomly sampled two latrine squat holes from each. Whenever possible, one superficial (depth up to 0.2 m) and one deep (depth 0.5 m - 1 m) fecal sludge samples [33] were collected for each latrine squat hole following the SOPs for fecal sludge sample collection and analysis (S1 Text).

The sample volume was set at 1-1.5 L of fecal sludge based on the capacity of our sampling devices. The actual sample volumes ranged from 0.5 L to 1.3 L due to the operational feasibility of sampling from the pit latrines. Environmental and personal safety were maintained at all steps of the fecal sludge sample collection procedures. A total of 40 (24 superficial and 16 deep) fecal sludge samples were collected and transported to Jimma University Molecular Biology and NTD Research Center using a cooler box. Once arrived at the laboratory, each fecal sludge sample was repeatedly processed (up to four sample preparations per collected fecal sludge sample) as described in previous sections, including the assessment of the TS. All results for the field evaluation were expressed as egg counts per gram of TS (EPG_TS_) for each of the STH separately.

#### Health and biosafety.

During the fecal sludge sample collection, transportation, processing, examination and storage, samples were presumed to contain pathogenic organisms. Accordingly, comprehensive biosafety persuasions recommended by U.S. Environmental Protection Agency (EPA) [34] were implemented. These measures included protection of fecal sludge sampling personnel and the surrounding environment at collection sites, as well as strict adherence to good laboratory practices for fecal sludge handling personnel, laboratory bench management, and safe disposal of fecal sludge residues.

### Statistical analysis

#### Analytical performance of the modified Fluke Catcher method.

We evaluated the performance of the modified Fluke Catcher method based on (i) the analytical sensitivity (the number of spiked *Ascaris* eggs that resulted in a positive test result with a probability of at least 95%) and (ii) the egg recovery rate. For both parameters, we used a regression model for count data including a random intercept for processed fecal sludge samples to account for correlation of counts within the sets of four repeated slides. To let coefficients represent the (logarithm) of the relative difference in expected and observed counts (i.e., the egg recovery rate), we included an offset for the natural logarithm of the expected number of eggs in a slide. As fixed effects, we considered the fecal sludge type (liquid, slurry, or semi-solid) and the number of spiked eggs (50, 100, 200, or 400) as categorical predictors. The final selection of fixed effects was based on the Akaike Information Criterion (AIC), where we considered an improvement in AIC as indicative of better model performance. Based on this model, for each fecal sludge type and range of number of spiked eggs, we calculated the analytical sensitivity in terms of the probability of detecting at least one egg (averaged over all the estimated random intercepts). The model was implemented in R (version 4.5.1), using the package *glmmTMB* (version 1.1.13). The script for the analysis is provided at https://gitlab.com/luccoffeng/echolatrine.

#### Proportion of positive slides and density of STH life stages in field samples.

We graphically explored the proportion of positive slides (%) and density (EPG_TS_) of STH life stages in fecal sludge samples across schools, depth of sampling (deep *vs.* superficial), and usage (boys *vs.* girls). For this, we determined (i) the proportion of positive slides (%) and the corresponding 95% confidence interval (CI) based on the binomial exact test and (ii) the median and interquartile range of the density (expressed in EPG_TS_) for each of the three factors.

#### Sources of variation in egg counts across field samples.

To assess and quantify the contribution of different sources (e.g., schools, latrine squat holes, sample preparations, repeated slides, and microscopists) to the variation in raw slide level egg counts in fecal sludge samples, we adapted an existing model for variance decomposition analysis of *S. mansoni* egg counts in humans [35]. With this model, we decomposed the variability of egg counts across sources using a multi-level Poisson regression model for all three species combined, using fixed effects to capture differences between schools, species, sex-specific latrine squat holes, and sampling depth. Fecal sludge type was not included as a predictor as it was always the same within five of the six schools. To correct for variation in absolute egg counts due to variation in consistency of stool samples, we included an offset for TS of each sample processed; consequently, all fixed effects represent the (logarithm) of the relative difference between samples in terms of EPG_TS_. We used gamma-distributed random effects to capture variation in egg counts between pit latrine squat holes, repeated sample preparations and microscopists.

In a sensitivity analysis, instead of gamma distributions we used the lognormal distribution for random effects. The contribution of pit latrine squat holes, repeated sample preparations and microscopists (i.e., the random effects) to the overdispersion of egg counts within schools was expressed in terms of the coefficient of variation or CV of each random effect (see **Info S2** for technical details).

The regression model was implemented in a Bayesian framework, using the probabilistic programming language Stan (cmdstan version 2.35.0) and the R package *cmdstanr* (version 0.9.0.9). Model parameters were sampled using 4 Markov chains, each with 1,000 warm-up and 1,000 sampling iterations, such that the effective number of samples from the posterior was at least 500 for all parameters. A description of the regression model in terms of equations is provided in **Info S2**. The corresponding R code to perform the analysis is provided at https://gitlab.com/luccoffeng/echolatrine.

#### Determination of the sampling and the analytical strategy to reliably assess the density of STH eggs in pit-latrines.

Before we embarked in a large-scale study to school-level estimates based on testing of fecal sludge *vs.* stool samples from children, we wanted to determine the optimal sampling and analytical strategy to reliably assess the density of STH eggs in pit-latrines. For this, we defined the reliability of latrine-based estimates of egg density levels in terms of the CV of the estimated school-level mean EPG_TS_ and compared this to the expected CV of stool results from children based on a simulation exercise. Here, we assumed that, like in our field study, the sample collection from latrines would not be affected by heavy dilution from, for instance, rainwater or flooding.

Egg counts in fecal sludge samples were simulated using the framework described above and in **Info S2**, quantified based on the spiking experiment and field data. Egg counts in stool samples from children were simulated using a similar and previously published simulation framework to determine the most cost-efficient survey design to make accurate program decisions [35]. In the present study, we assumed that 52 children would be sampled and tested; this sample size was based on the results of Kazienga A., *et al*. [36] who determined that this sample size allows for reliable decision making. Simulated survey results for school pit latrines and children were both summarized in terms of the expected relative variability (expressed in CV) of the estimated school-level mean EPG_TS_ and eggs per gram of stool (EPG), respectively. These school-level CVs were simulated and calculated for a range of assumed true infection levels in the school. By necessity, we assumed that true EPG_TS_ in fecal sludge is the same as the true mean EPG in stool from children. As for the observed egg counts in simulated samples, we assumed that the egg recovery from latrine samples was equal to estimated egg recovery of the Fluke Catcher method in the egg spiking experiment. For Kato-Katz based on stools from children we assumed that the egg recovery was either 100% (i.e., higher than the Fluke Catcher method) or 50% (sensitivity analysis). These assumptions will need to be revisited in future studies that sample both children and latrines from the same schools. The code to conduct the simulation experiment can be found at https://gitlab.com/luccoffeng/echolatrine.

## Results

### Field evaluation of fecal sludge sampling devices

**Fig 3:**
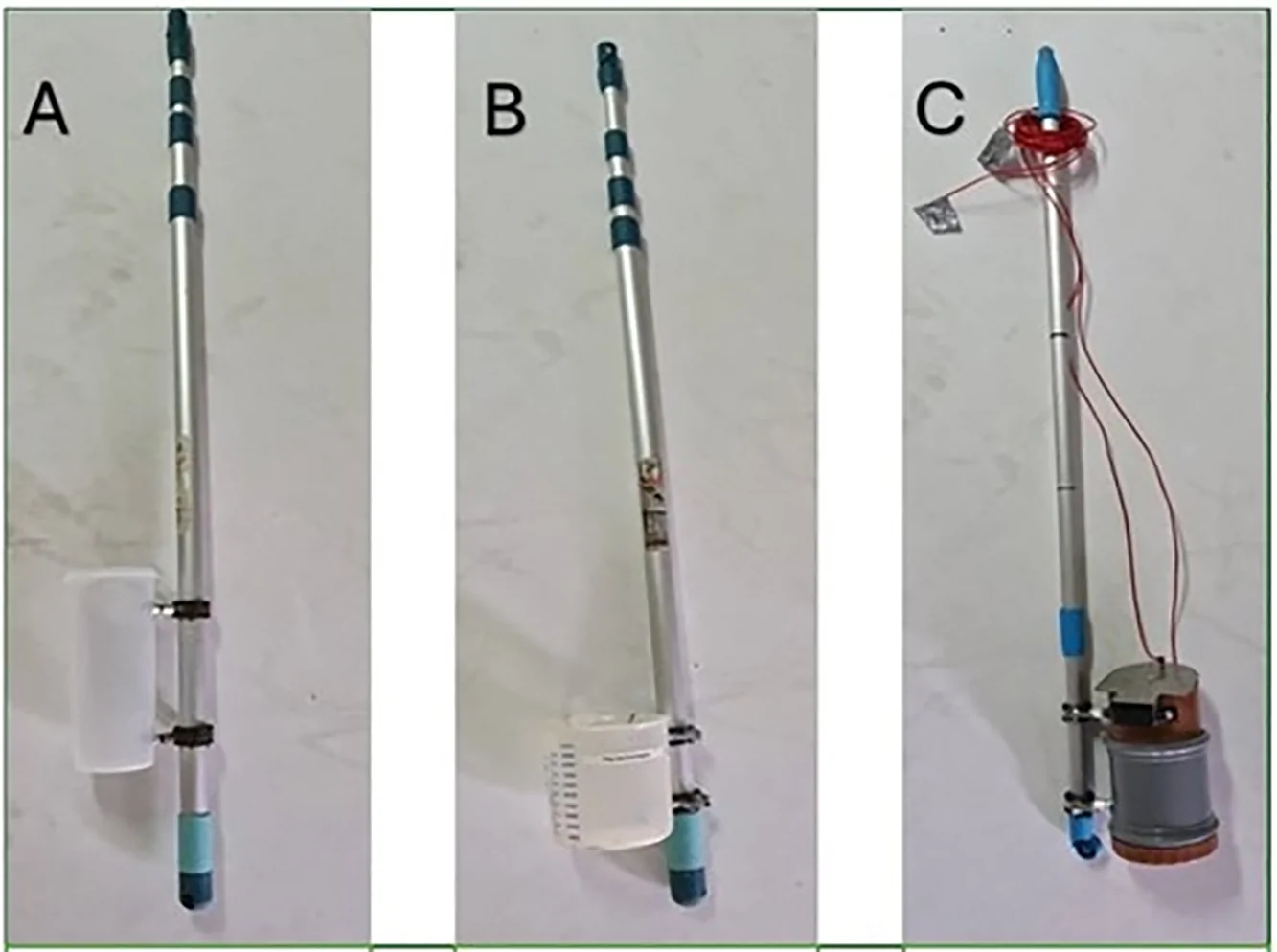
In-house developed fecal sludge sample collection devices. **Panels A** and **B**, represent devices with an open sample container with different diameters (Panel A: 9 cm and Panel B: 10 cm). **Panel C** represents a sampling collection device that could be opened/closed by pulling a string, the diameter of the container equals 10 cm.

The exploratory search of the scientific literature and targeted Google searches identified a range of self-made, commercially available, and modified commercial devices for fecal sludge sampling [36–44]. Based on the insights gained from this search, we identified two key criteria: (i) low production cost and (ii) feasibility of local manufacture using readily available materials. Based on this, we designed three prototype devices for *in situ* grab sampling (Fig 3). The design accounted for variation in pit latrine squat hole sizes, pit depths and fecal sludge types (liquid, slurry and semi-solid). Each device had a retractable stick (pole) with a maximum length of 5 m that allowed sampling at required depth, and a container with a capacity of 1 to 1.5 L was attached to one end of the stick. Two devices consist of an open sample container with diameters of 9 cm (Fig 3A) and 10 cm (Fig 3B). The third device used a closed container with a diameter of 10 cm, and its lid could be opened or closed by pulling a string (Fig 3C) to collect samples at a specific depth within a pit latrine.

During field evaluation, the following main operational challenges were identified: narrow squat holes, the presence of foreign objects (e.g., plastic waste) within pit latrines, dried or solid fecal sludge, and fecal sludge at greater depths. Based on these field observations, the prototypes were modified for use in the remainder of the study. To accommodate narrow squat holes, a narrower sampling container was secured with plastic cable ties. To enable sampling from deep pit latrines (>4 m), an additional extension stick was added and secured with duct tape. Despite these modifications, the devices remained unsuitable for sampling dried or solid fecal sludge and for latrines with insufficient sludge volume.

### Analytical performance of the modified Fluke Catcher method

The TS of the two collected fecal sludge samples were 21.5% and 17.9%. Sub-samples of these fecal sludge samples were diluted with tap water to generate three slurry (TS = 7.7%, 9.7% and 10%) and three liquid (TS = 2.6%, 3% and 4.8%) fecal sludge samples. In total eight samples were spiked and analyzed using our modified Fluke Catcher method. Variability of egg counts in repeated slides closely followed a Poisson distribution (Fig A in S2 Text). A numeric summary of the experiment is provided in (Table A in S2 Text).

**Fig 4:**
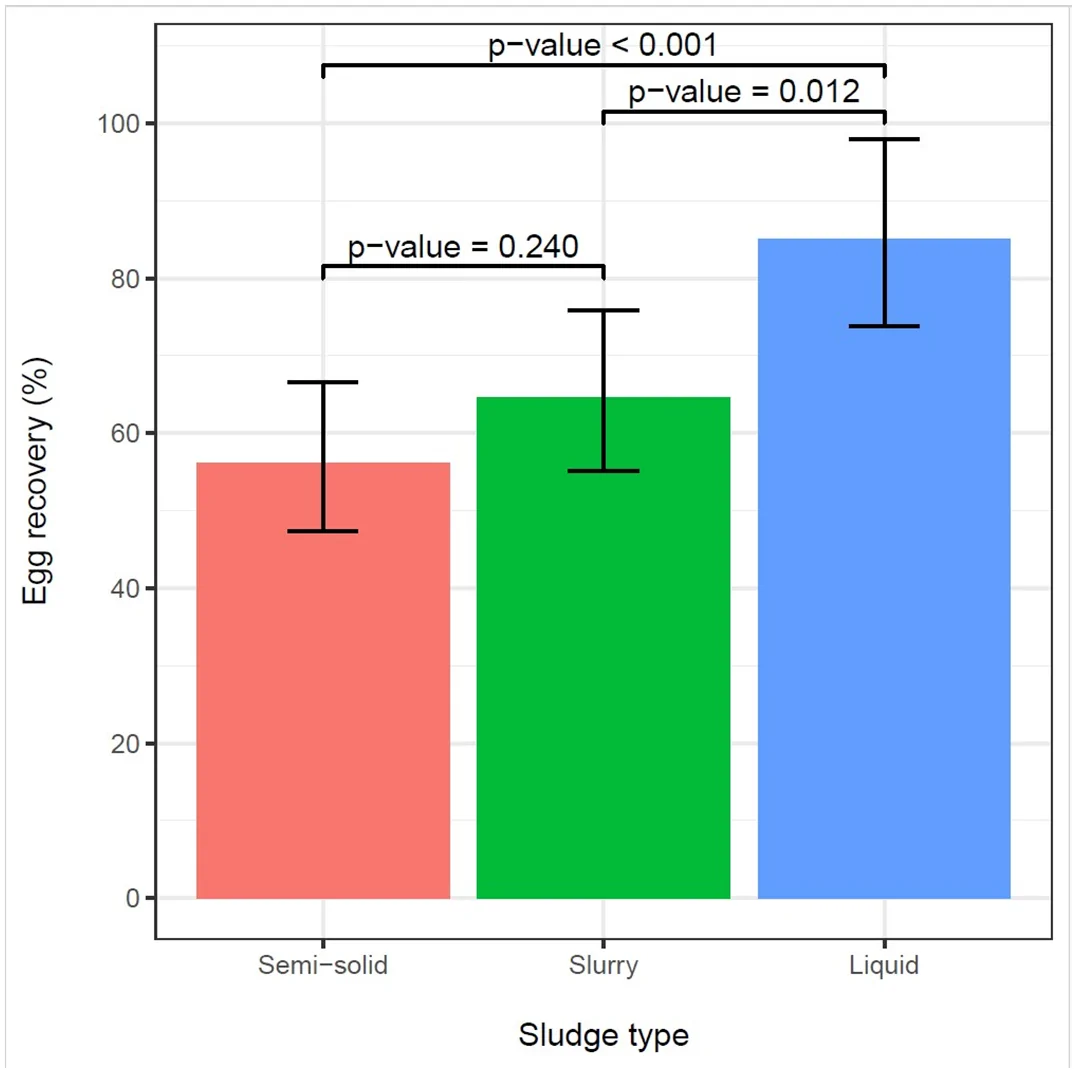
Estimated egg recovery rate from fecal sludge spiked with known quantities of Ascaris eggs. Each bar represents the average egg recovery rate across the fecal sludge types that were spiked with 50, 100, 200 or 400 eggs. Error bars indicate the 95% confidence interval around the point estimates. Results are based on a Poisson regression model with a random intercept per fecal sludge sample (from which four repeated slides were taken), an offset for the natural logarithm of the expected number of eggs, and a fixed effect for fecal sludge type.

Overall, the recovery rate of *Ascaris* eggs by our modified Fluke Catcher method was significantly higher in liquid sludge (85%, 95% CI: 74%–98%) than in slurry (65%, 95% CI: 55%–76%) and semi-solid sludge (56%, 95% CI: 47%–67%) (Fig 4). The egg recovery rate did not differ significantly between the slurry and semi-solid sludge. The number of spiked eggs was not a significant predictor of the egg recovery rate, either on its own (AIC = 1,068.4), or in combination with sludge type (AIC = 1,058.6), or as an interaction with sludge type (AIC = 1,067.0).

**Fig 5:**
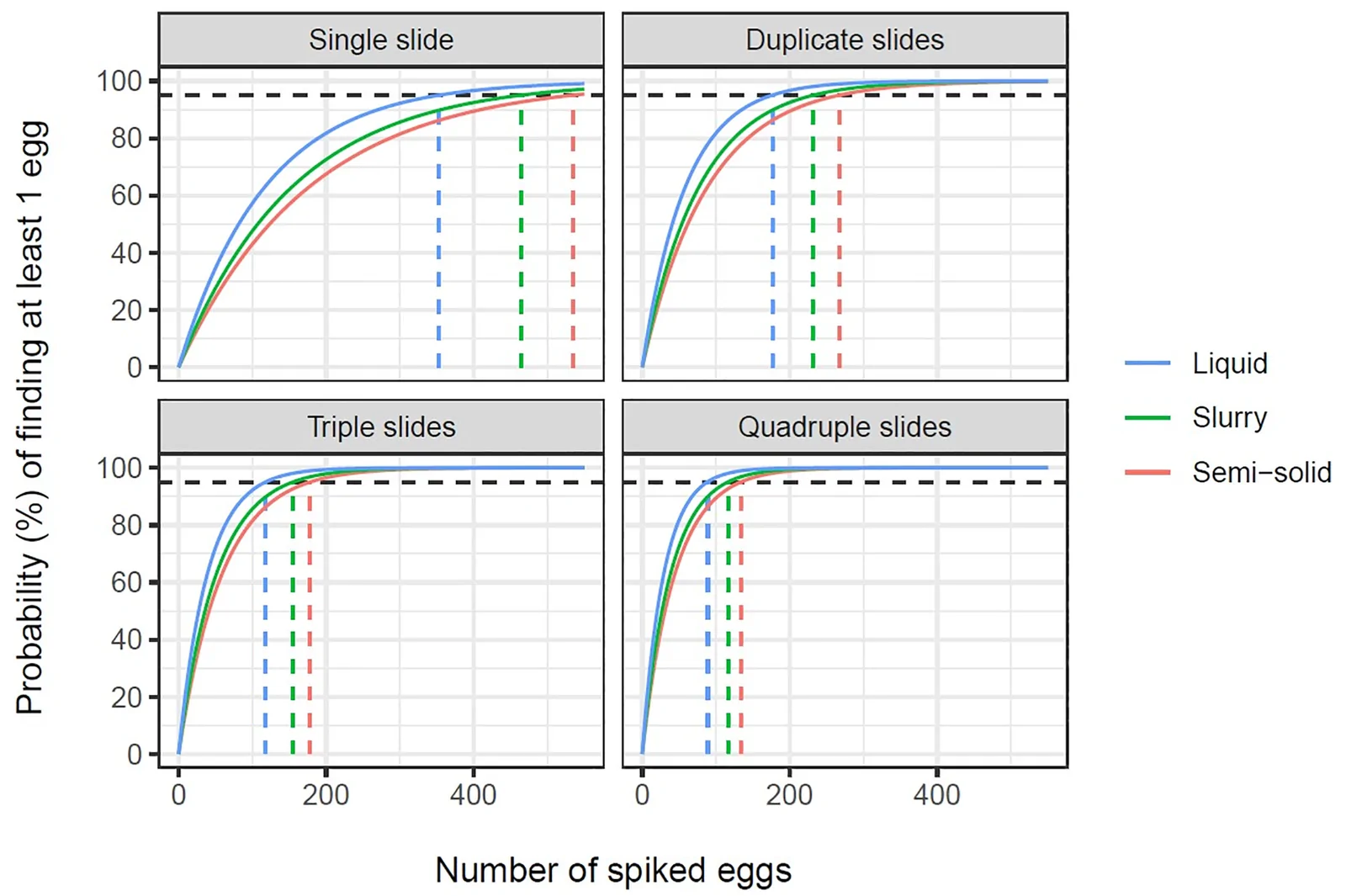
The analytical sensitivity of the modified Fluke Catcher methods to detect Ascaris eggs in fecal sludge samples. This represents the predicted probability of finding at least one egg for a wide range of spiked eggs for three fecal sludge types and different numbers of slides (each of 50 µL).

Based on the estimated egg recovery rate, we calculated the analytical sensitivity of the Fluke Catcher method in terms of the probability of finding at least one egg (Fig 5). Generally, when four slides (each 50 µL) were examined per sample, the probability of finding at least one egg was ≥ 95% (dashed horizontal black line in Fig 5) when ≥90 eggs were spiked for liquid samples, ≥ 116 eggs for slurry samples, and ≥135 spiked eggs for semi-solid samples (dashed vertical lines). When reducing the number of slides, the analytical sensitivity dropped for all three fecal sludge samples.

### Proportion of positive slides and density of STH life stages in field fecal sludge samples

A total of 352 slides (each 50 µL) were prepared from 88 sample preparations (each 10 g of fecal sludge sample), obtained from 40 samples collected at varying depths in pit latrines across six schools (Table 1). The consistency of fecal sludge samples varied across latrines and holes, with most samples being of the semi-solid type (n = 28) and the remainder being either slurry (n = 4) or liquid (n = 8). Superficial and deep fecal sludge samples from the same pit latrine squat hole were always of the same consistency type.

**Table 1:** Overview of collection and processing of fecal sludge samples from 8 pit latrines across 6 schools.

School	Number of sampled pit latrines	Pit latrine type	Total number of squat holes sampled	Sampling depth	Depth of deep samples (cm from sludge surface)	Repeated number of sample preparations of 10 g	Number of repeated slides	Total number of slides
Dilfire	1	Common	4	Superficial + deep	30–65	1 or 4*	4	16 + 64*
Ginjo	2	Sex-specific	4	Superficial	–	1	4	16
Hirmata	2	Sex-specific	4	Superficial + deep	30–85	4	4	128
Jimma	1	Common	4	Superficial	–	4	4	64
Kito	1	Common	4	Superficial + deep	70–100	1	4	32
Mendera	1	Common	4	Superficial + deep	50	1	4	32
**Total**	**8**	**–**	**24**	**–**	**30–100**	**–**	**–**	**352**

* For two of the four holes (one for each sex), only a single sample preparation of 10 g of fecal sludge was processed per sampling depth, leading to 16 slides (2 squat holes x 2 depths x 1 sample preparation x 4 slides); for the other two squat holes (one per sex), four repeated sample preparations of 10 g of fecal sludge were processed per sampling depth, leading to 64 slides (2 squat holes x 2 depths x 4 sample preparations x 4 slides).

**Fig 6:**
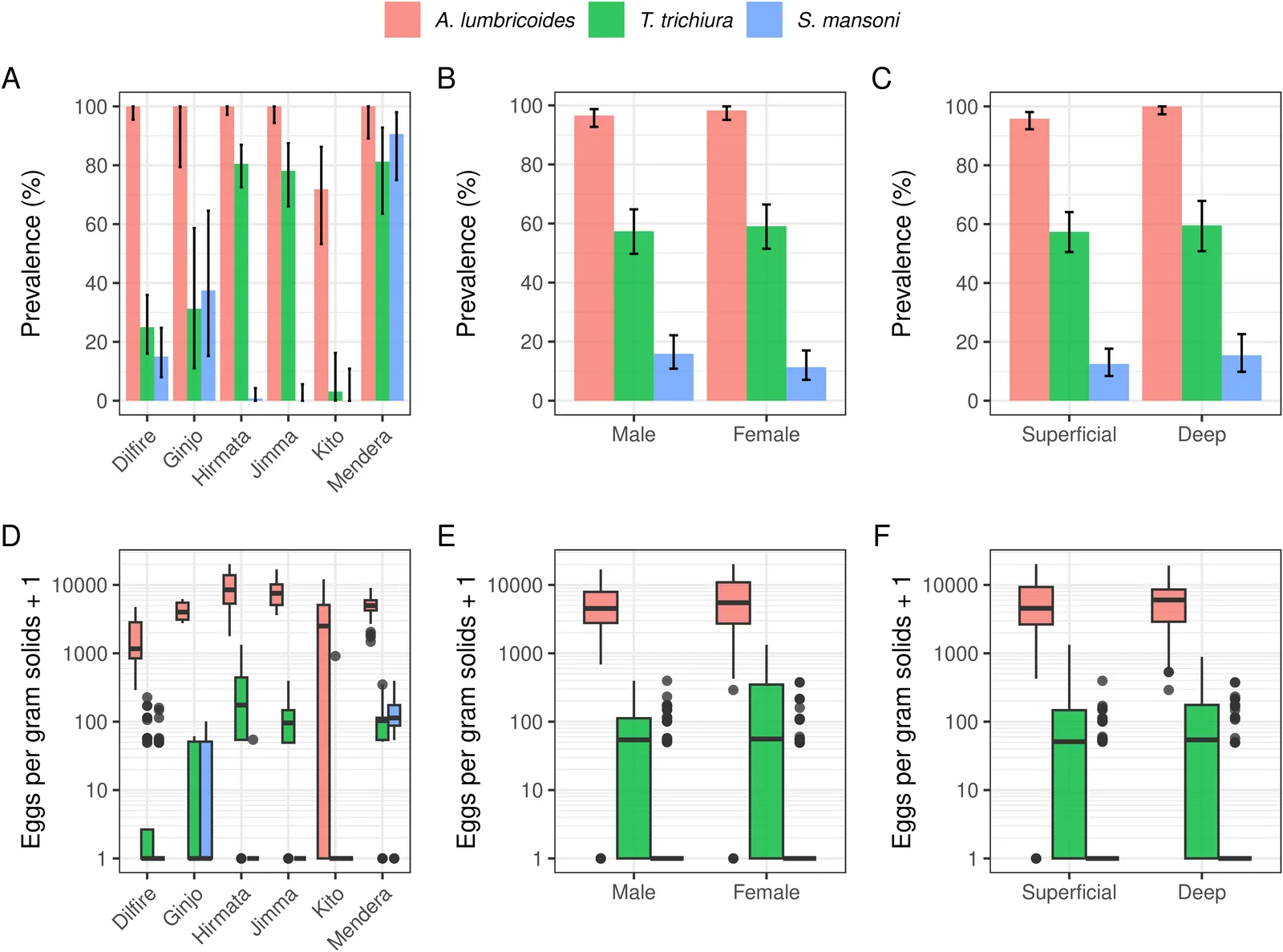
Proportion of positive slides (Prevalence) and density of helminth eggs across 352 slides from six schools. The top row represents the prevalence (proportion of positive slides) of A. lumbricoides, T. trichiura and S. mansoni eggs across 6 schools (**Panel A**), sex-specific latrines (**Panel B**), and the depth at which samples were collected (**Panel C**). The boxplots in the bottom row represent the egg density (eggs per gram total solids detected in 50 µL of sample preparation) across schools (**Panel D**), sex-specific latrines (**Panel E**), and the depth at which samples are collected (**Panel F**). Boxplots represent the median (thick horizontal line) and the first and third quartiles (lower and upper bound of each box). The whiskers (thin vertical lines) extend from the first or third quartile to the lowest or largest value, respectively, no further than 1.5 times the interquartile range. Data beyond the end of the whiskers are plotted individually (black bullets). Note that the y-axes of panels **D-F** are logarithmic and represent the egg density plus one (to include zero values in the plot). Further, note that some of the boxplots for S. mansoni do not show blue because the median and first and third quartiles are all the same (horizontal thick black line segments) due to the high number of zero egg counts.

Across the 88 fecal sludge sample preparations (352 slides), we observed several helminth life stages of medical importance, including *Ascaris, Trichuris*, *Schistosoma mansoni* and others such as *Hymenolepis nana*, *Enterobius vermicularis,* and *Taenia* spp. We also observed hookworm-like eggs and unknown larvae but did not use any molecular tools to further identify these. *Ascaris* eggs were the most prevalent (343/352 slides; 97%) and found at the highest density (6,240 mean EPG_TS_, including egg-negative slides). *Trichuris* eggs were present in just over half of the slides (205/352; 58%) and in a lower density (133 mean EPG_TS_). *S. mansoni* eggs were the least prevalent (48/352; 14%) and found at the lowest density (17 mean EPG_TS_). Summary of the proportion of positive slides and density of *Ascaris*, *Trichuris* and *S. mansoni* eggs in the fecal sludge across six schools is available in S2 Text – Table B. Proportion of slides positive for *Ascaris* was 100% across five of the schools’ latrines, and 72% in one school latrine (Kito; Fig 6A). Proportion of slides positive for *Trichuris* eggs in slides varied considerably across schools’ latrines (ranged 3%–80%), as did the proportion of slides positive for *S. mansoni* eggs (0%-91%). For all three helminth species, the egg density varied considerably across schools’ latrines, even when the proportion of positive slides was 100%, as for *Ascaris* (Fig 6D). However, for all three species, the proportion of positive slides and density of eggs did not seem to vary much between boys’ and girls’ latrines (Fig 6B and 6E) or superficial and deep fecal sludge samples (Fig 6C and 6F).

### Sources of variation in helminth egg counts across field fecal sludge samples

To inform the structure of the count regression model, we visually assessed the level of overdispersion in slide level egg counts (variance relative to the mean). Overdispersion of egg counts was the strongest across pit latrine holes from the same school, with the variance across holes consistently exceeding the school-level mean egg count (S2 Text – Fig B). In contrast, the variance in egg counts was very similar to the mean count across slides and across repeated sample preparations (S2 Text – Fig B). The only exception was *Ascaris*, for which the variance tended to exceed the mean count for the higher egg counts. A visual inspection of the raw slide-level count data suggested that intra-operator variability might have contributed to overdispersion in slide-level egg counts for *Ascaris*. Out of each set of four repeated slides, the two highest and two lowest counts were often performed by different microscopists (i.e., non-overlapping counts). Of the 88 sets of four repeated slides, 80 sets were read by two microscopists. Within these 80 sets, the frequency of non-overlapping counts by the different microscopists was significantly higher than the expected 33% (1/3): 54% for *Ascaris* (binomial exact *p*-value <0.001), indicating that the presence of more than one microscopist significantly contributed to variation in egg counts. A similar pattern was found for *Trichuris* (64%, *p*-value <0.001) and *S. mansoni* (86%, *p*-value <0.001). Based on these observed patterns in variability of egg counts, we specified a multi-level count regression model with random effects capturing variation between holes, samples, slides, and microscopists, as described in the methods section. As only 5 microscopists were involved in the counting of eggs, and none of them seemed to systematically over- or underestimate counts across the sets of 4 slides (each 50 µL), the random effect for microscopist was assumed to be independent across different sets of slides.

For all three parasite species, the regression model indicated that egg counts did not vary notably between squat holes for boys and girls: the relative differences in parasite-specific EPG_TS_ in pit latrines for girls *vs.* boys (reference) were 1.1 (*Ascaris*; 95% Bayesian credible interval (BCI): 0.68–1.55), 0.87 (*Trichuris*; 0.5–1.4), and 0.7 (*S. mansoni*; 0.3–1.3). Sex was therefore not retained in the model as a predictor. Sampling depth was kept in the final model (S2 Text – Fig C), as *Ascaris* eggs density was 42% higher in deep fecal sludge samples than in superficial samples (95% BCI: 21%–66%). *Trichuris* and *S. mansoni* egg density also tended to be higher at deeper sampling depths, although the 95% BCI of these differences did span 0% (*Trichuris*: 11%, 95% BCI: -13%–38%; *S. mansoni*: 63%, 95% BCI: -3%–150%). Across all three helminth species together, deep fecal sludge samples contained 34% more eggs (95% BCI 18%–53%) than superficial samples. The association between average and variance of egg counts across semi-solid and slurry samples in field settings is also summarized in Fig D in S2 Text.

The model further confirmed that within schools’ latrines and per species, the overall level of overdispersion in egg counts (${CV}_{total}=\text{0}.\text{7}\text{2}$) was mostly driven by variation between pit latrine squat holes (${CV}_{J}=\text{0}.\text{5}\text{6}$). Variation between sample preparations of 10 g was the second most important source of variation (${CV}_{S}=\text{0}.\text{3}\text{3}$) and variation between microscopists was the least important (${CV}_{M}=\text{0}.\text{1}\text{8}$). Table 2 summarizes these findings. This pattern held in a sensitivity analysis using a multi-level Poisson model with log-normal-distributed random effects (${CV}_{total}=\text{0}.\text{8}\text{1}$, ${CV}_{J}=\text{0}.\text{6}\text{5}$, ${CV}_{S}=\text{0}.\text{3}\text{4}$, ${CV}_{M}=\text{0}.\text{1}\text{8}$).

**Table 2:** Model-estimated parameter values for variance decomposition of egg counts in fecal sludge samples from school pit latrines.

	Gamma-distributed random effects			Log-normal-distributed random effects		
Parameter	Posterior mean	95% Bayesian credible interval		Posterior mean	95% Bayesian credible interval	
***Mean EPG***_***TS***_ for A. lumbricoides*						
Dilfire	2,501	1,439	4,142	1,943	1,136	3,061
Ginjo	5,179	2,975	8,577	4,519	2,479	7,304
Hirmata	8,418	5,207	13,126	6,926	4,065	10,751
Jimma	8,661	5,336	13,664	7,665	4,630	11,875
Kito	3,111	1,773	5,064	2,489	1,374	3,985
Mendera	4,727	2,780	7,547	4,204	2,421	6,707
						
**Mean EPG**_**TS**_ for T. trichiura*						
Dilfire	26	14	45	18	9	32
Ginjo	31	13	62	26	11	50
Hirmata	254	153	401	158	92	249
Jimma	110	65	174	92	53	147
Kito	60	17	136	53	15	115
Mendera	114	63	190	97	53	159
						
**Mean EPG**_**TS**_ for S. mansoni*						
Dilfire	12	6	21	10	5	18
Ginjo	29	12	53	24	10	46
Mendera	107	58	182	93	49	151
						
**Relative difference between deep versus superficial samples**						
*A. lumbricoides*	1.42	1.21	1.66	1.43	1.22	1.65
*T. trichiura*	1.11	0.87	1.38	1.11	0.86	1.39
S*. mansoni*	1.63	0.97	2.50	1.62	0.97	2.48
						
**Coefficient of variation (CV)**						
Total CV	0.72	0.61	0.85	0.81	0.66	1.00
CV for holes	0.56	0.45	0.70	0.65	0.49	0.85
CV for samples	0.33	0.27	0.39	0.34	0.28	0.42
CV for microscopists	0.18	0.15	0.21	0.18	0.15	0.22

*Mean egg count per gram total solids (EPG_TS_) in superficial fecal sludge samples. Estimates from the gamma-distributed random effects model represent arithmetic means, whereas values for the log-normal-distributed random effects model represent geometric means. Average EPG_TS_ for *S. mansoni* were only estimated for three schools (Dilfire, Ginjo, Mendera) as no eggs were detected in other schools (Jimma, Kito) or only in a single sample (Hermata).

### Fecal sludge sampling strategy to reliably assess the density of STH eggs in pit latrines

**Fig 7:**
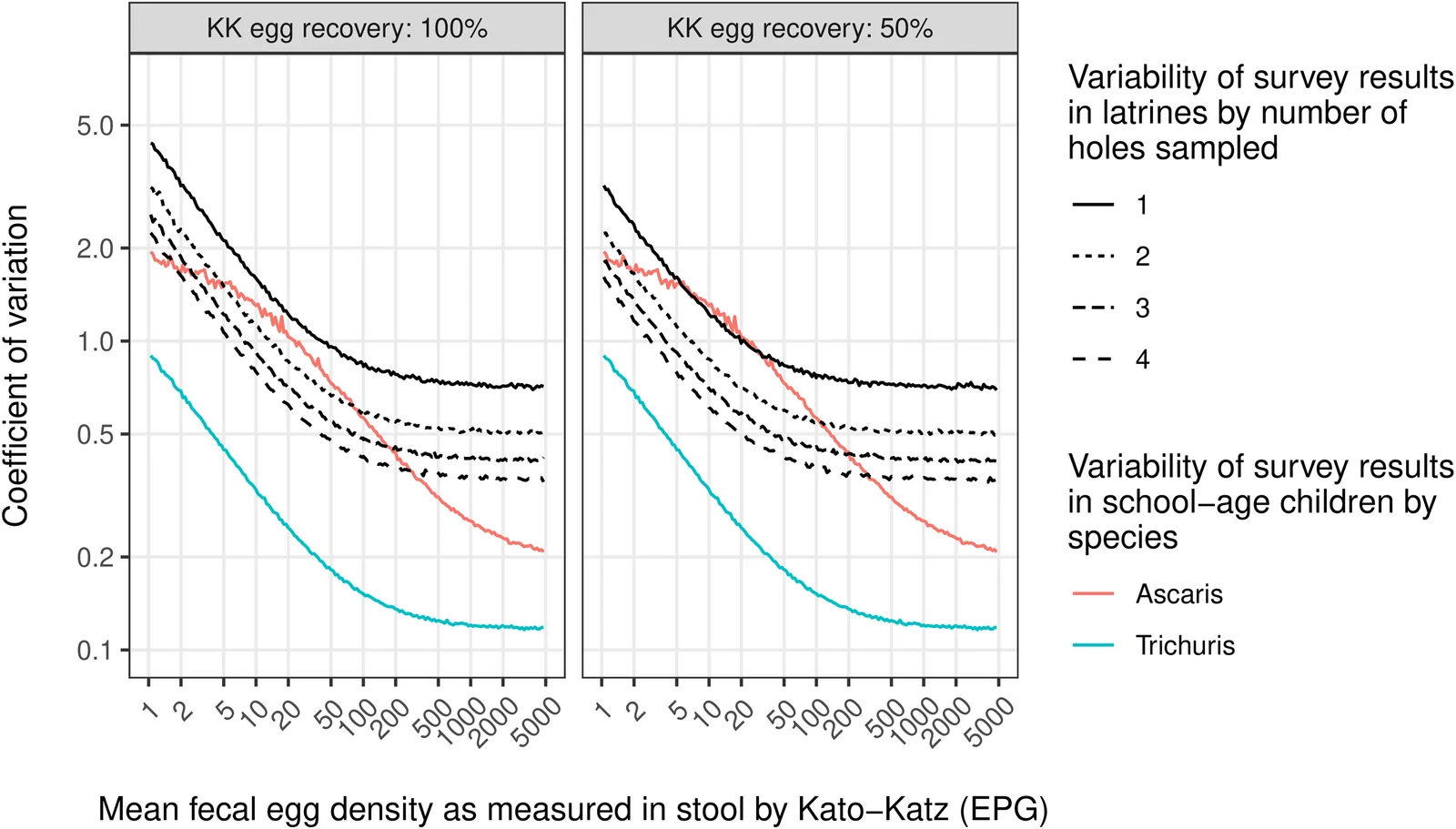
Association between average and variance of egg counts across slides, processed samples, and pit latrine holes in field settings. Variability of survey results is expressed in terms of the coefficient of variation of the school-level mean egg count across repeated survey results (y-axis), based on 10,000 repeated simulations. Survey results in school-age children are based on duplicate Kato-Katz thick smear (KK), using a single well-homogenised stool sample per child, adopting a previously developed simulation framework for soil-transmitted helminths in school-age children [36] and assuming either 100% (better than the Fluke Catcher method) or 50% egg recovery (comparable to the Fluke Catcher method). Fecal sludge testing with the Fluke Catcher method was assumed to recover 50% of the eggs (based on the spiking experiments; Fig 4).

We determined an optimized fecal sludge sampling strategy based on the association between the variability in measured school-level average egg density (expressed as CV) and the true school-level mean egg density (a proxy of endemicity) for different scenarios of diagnostic effort (number of sample preparations) and sampling effort (number of squat holes sampled). We are assuming one slide per sample preparation. Fig 7 illustrates two aspects of this association (black lines).

First, the CV changes with endemicity. The variability is highest when the mean egg density is lower, then steeply drops as a function of increasing true mean egg density, after which it stabilizes from 50 EPG onwards (as measured in stool samples with Kato-Katz thick smear). Second, when aiming to reduce the variability in survey results it is better to sample more squat holes than to repeatedly process the same fecal sludge sample. For instance, assuming a mean egg intensity of 100 EPG (as measured in stool, assuming 100% egg recovery by Kato-Katz thick smear), the CV of EPG_TS_ in latrine fecal sludge was 0.85 when surveys are based on a single slide from a single fecal sludge sample and squat hole. Increasing the number of sampled squat holes to four (still single sample preparation and one slide per sample preparation) reduced the CV to 0.42, whereas increasing the number of sample preparations to four (still single slide per sample preparation) only reduced the CV to 0.63. These findings are directly explained by the finding that variability in egg counts in the field study (previous section) were mostly explained by variability between squat holes (i.e., highest CV, Table 2). Fig 7 shows that these patterns remained qualitatively the same if the egg recovery of Kato-Katz thick smear was assumed to be 50% (i.e., comparable to that off the Fluke Catcher method) instead of 100%.

Generally, the results of stool-based surveys among children (red and blue lines in Fig 7) were less variable compared to latrine-based surveys. However, the CV of latrine-based surveys did not exceed that of stool-based surveys for *Ascaris* when the mean fecal density was below 100 EPG (as measured in stool by Kato-Katz thick smear) and at least 3–4 squat holes were sampled. For detection of *Trichuris* eggs, surveys among children yielded consistently more precise results (lower CVs) than latrine-based surveys. If the egg recovery of Kato-Katz thick smear was assumed to be 50% instead of 100%, the accuracy of the EPG_TS_ based on fecal sludge became more similar to Kato-Katz-based EPG (i.e., the black lines in the right panel of Fig 7 are shifted to the left by a factor 2 compared to the left panel).

## Discussion

### Fecal sludge sampling devices can be locally made

Designing and field testing of prototypes for fecal sludge sampling from school pit latrines showed that, simple devices can be constructed from readily and locally available materials and adapted to accommodate variable pit latrine structures. The earlier prototype devices we designed faced several operational challenges that required ad-hoc modifications, including narrower fecal sludge sampling containers and extended sampling poles (stick), which remained feasible under field conditions. The modified fecal sludge sampling prototypes were generally successful, except for a few pit latrines with dried/solid fecal sludge types and insufficient fecal sludge volume (e.g., newly constructed, recently emptied and infrequently used school pit latrines). These limitations indicate that latrine-based survey might not be universally applicable across all sanitation contexts.

### Our modified Fluke Catcher method is simple, cheap and has moderate performance

To develop a streamlined fecal sludge sample processing, we reviewed existing protocols for the detection and quantification of STH eggs from fecal sludge samples. Although several methods have been described [30,36,37,45,46], many rely on labor-intensive procedures, expensive equipment, and/or reagents/solutions which are often expensive or toxic for both humans and environment. In the present study, we combined the Fluke Catcher with a 20 µm pore size sieve for the detection and quantification of STH eggs in fecal sludge, and by doing so omitting the need of chemical solutions [25,30]. The cost of the modified Fluke Catcher is estimated to be 478.50 EUR, which is 94% less expensive than that of our previously described egg-count method to detect and quantify STH in soil samples (8,000.00 EUR) [25]. The egg recovery rate depended on the consistency of the fecal sludge, with more eggs being recovered in liquid fecal sludge (85% *vs.* slurry (65%) *vs.* semi-solid fecal sludge (56%)). The evaluation of the performance of the modified Fluke Catcher method showed that the analytical sensitivity ranged from 90 to 135 *Ascaris* eggs, the performance being highest when four slides were examined and when eggs were spiked in liquid fecal sludge samples and being lowest when one slide was examined and when eggs were spiked in semi-solid samples. The observed differences in egg recovery rate and analytical sensitivity across fecal sludge consistency may be explained by the ‘sticky’ nature of *Ascaris eggs*, which can easily get trapped in denser fecal sludge and therefore get lost in semi-solid compared with more liquid samples [47,48]. A review study on recent advances in quantification of STH eggs from environmental samples also reported similar egg recovery rates (57 – 80%) [24], indicating that our egg counting method has a moderate performance. Of course, we only conducted experiments with *Ascaris* eggs (we could obtain it in large quantities), and thus extrapolation on the performance of our modified Fluke Catcher method to other STH and helminthiases should be done with care. For example, a potential difference in recovery efficiency for other STHs might be expected. Eggs for *Trichuris* are smaller compared to *Ascaris* (egg dimension: fertilized *Ascaris* 45–75 x 35–50 μm vs. *Trichuris* 50–58 x 20–27 μm) [49], and hence eggs might be lost. For hookworms, we might even miss relevant life stages. In contrast to *Ascaris* and *Trichuris*, for which the infectious larval stage does not hatch, we expect to find larval stages in the fecal sludge for the hookworms. These stages are much larger than the eggs (larvae dimension: rhabditiform (L1) 250–300 x 15–20 µm) [49], and thus will be withheld on the sieves of the Fluke Catcher, resulting in an underestimation of the density of hookworms. In addition, the method has some important limitations. The need for a centrifuge is one of them. Exploring alternatives such as passive sedimentation would therefore be recommended [24]. A potential tool that could assist in this is the FECPAK^G2^ sedimenter, which has already been optimized for human stool by Ayana *et al*. [31]. Similarly, our fecal sludge sample processing protocol is an open system, meaning that further methodological modifications, such as adoption/adaptation of FECPAK^G2^ sedimenter or other closed system designs, are required to transform it into a closed system to minimize biosafety risks [50–52].

### Variation in school helminth egg density underscores the potential of a latrine-based survey

We observed six medically important helminths (*Ascaris, Trichuris*, *S. mansoni*, *E. vermicularis*, *H. nana* and *Taenia* spp.). We also detected hookworm-like eggs, but in absence of any molecular analysis, we did not feel comfortable in drawing conclusions on the presence of this STH. Noteworthy is the observed variation in density (mean EPG_TS_) across schools for *Ascaris*, *Trichuris* and *S. mansoni*. Similarly, historical data on a stool-based survey across 10 schools in the same study area (2015 and 2018; [32]) indicated a wide range in school prevalence and infection intensity for each of the STH (see Table C in S2 Text). Fig E in S2 Text plots the historical prevalence data with the density in fecal sludge samples for six schools involved in the present study, underscoring the potential of a latrine-based survey. In a follow-up study we will conduct a head-to-head comparison of a latrine-based survey (mean helminth egg count in fecal sludge) and a stool-based survey (prevalence and intensity of helminth infections in schoolchildren) across 25 schools in Jimma Zone, Ethiopia.

### Insights into sources of variation essential to determine sampling and analysis strategy

To determine the sampling strategy (i.e., required number of squat holes per latrine, depth of sample collection) and the analysis of the fecal sludge samples (the number sample preparations and slides) for the follow-up study, the current study prioritized understanding the sources of variation in helminth egg counts. The main source of egg count variations was due to difference in egg counts between squat holes, followed by variation in sample preparations and microscopists. This pattern of better increasing sampling efforts over analysis efforts is in line with the study conducted by Koottatep *et al*. [41]. This study reported that, in a fecal sludge treatment plant, multiple sampling at different points provides more representative estimates than a single point sampling. This suggests that sampling additional locations within a latrine may further improve representativeness in highly heterogeneous fecal sludge within the same school pit latrine. In contrast, no significant differences in density were observed between boys’ and girls’ pit latrines. A significant difference in egg counts across the depths of sampling was observed for *Ascaris*, but not for the other helminths under research. Despite of these differences in egg counts (probably due to accumulation of eggs due to gravidity), we would continue collecting superficial samples only. This is because the superficial layer probably represents more recently deposited stool, and thus it is potentially more representative for the current status of STH contamination. The subsequent simulation study indicated that a latrine-based survey was only as precise as a stool-based survey for *Ascaris* and when the fecal egg density was below 100 EPG_TS_. For the detection of *Trichuris* eggs, surveys among children yielded consistently more precise results (lower CVs) than latrine-based surveys. This difference may reflect that the CV for *Trichuris* egg counts might be expected to be lower than what currently shown here, as our estimates of variability of latrine-based surveys are mostly driven by *Ascaris*. Also note that we have been focusing on the variability of surveys only. At this stage we did not consider the accuracy of the surveys nor the probability of correct program decision-making (continue or scaling down the frequency of deworming were warranted). This too, will warrant further research.

## Conclusion

This study demonstrates the design and feasibility of low cost locally manufactured devices for fecal sludge sampling in school pit latrines and provides a detailed evaluation of the analytical performance of an egg-counting method under laboratory conditions. It also identified the most important sources of variation in egg counts, allowing to define a more evidence-based sampling collection and analysis strategy. Together, the findings provide a methodological proof-of-concept. Future studies directly comparing latrine-based and stool-based surveys are now needed. For this, we suggest sampling at least three holes per pit latrine from top layer of fecal sludge, one sample processing and one slide (representing a volume of 50 µL).

## Supporting information

S1 Text SOP: Pit latrine fecal sludge sample collection and analysis. (DOCX)

S2 Text Theoretical framework to decompose sources of variation in egg counts. (DOCX)
