# Supplementary material for: Development, field-testing and optimization of tools to quantify soil-transmitted helminths in fecal sludge from school pit latrines in Ethiopia
Source: PLoS Negl Trop Dis. 2026 Sep 15;20(9):e0014724. doi: 10.1371/journal.pntd.0014724 (PMC13592699; doi:10.1371/journal.pntd.0014724)
Supplement: Supplementary file 1 [file pntd.0014724.s001.docx]

**S1 Text : SOP: Pit latrine fecal sludge sample collection and analysis**

# ***Purpose***

This Standard Operating Procedure (SOP) is used for the detection and quantification of ova of soil-transmitted helminths (STHs) and other intestinal helminthic parasites in latrine sludge samples. The SOP describes procedures of latrine sludge sample collection, transportation, processing and examination. Preparation of this SOP is one objective of development and validation of a latrine-based surveillance system to monitor STH control programs and to ensure early detection of anthelmintic resistance (AR).

# ***Materials, Reagents and Equipment***

- Plastic lab apron
- Veterinary plastic gloves
- Gloves
- Face mask
- Safety boots
- Torch light
- Latrine sludge sample collection devices
- Sludge sample container
- Hand shovel
- Plastic zipper
- Cold box
- Water-full plastic jerry can (20-25 L)
- Dish-washing brush
- Soap
- Disinfectants (75% alcohol, 5%–9% sodium hypochlorite)
- Lab coat
- Metal spatula
- Wooden spatula
- Digital weight scale
- Graduated plastic cups
- Tap water
- Fluke Catcher sieve sets
- Graduated plastic jug (3 litre)
- Sieve (20μm pore size, 100mm diameter)
- 50μm plastic conical test tube
- 50μm tube rack
- Centrifuge
- Pipette (20-200μl)
- Pipette tips
- Microscope slides
- Coverslips (20mm x 20mm)
- Microscope

# ***Procedures***

## A. **Fecal sludge sampling from pit latrine**

1. Pack all materials needed for the collection of latrine sludge samples using field work check list for latrine sludge sample collection.
2. Prepare all materials and devices needed for the collection of latrine sludge sample at the site of sludge sample collection.
3. Apply all needed personal protective equipment (safety boots, plastic lab apron, face mask, veterinary gloves, and gloves).
4. Identify and select latrine pit holes and inspect the internal contents using light torcher.
5. Insert sludge sample collection device through the latrine hole to the surface of the latrine sludge and record the latrine pit depth from the top slab.
6. Deepen the sludge collection device by moving up and down to the latrine sludge and check if the required sample amount is collected using torcher light.

**Note:** If sludge sample from different depth is required, close the collection device with the lid and open at the required depth by pulling the device string. Then, close the collection device after the sludge sample is filled in the collection device container.

7. Pull up the collection device containing the sludge sample and pour to the labelled container, apply plastic zipper, and keep in cold box for transportation.

**Note:** Apply all necessary personal and environmental decontamination after collection of sludge samples.

B. **Sludge sample processing and examination**
1. Wear lab coats, face mask and gloves, and prepare all materials needed using check list for sludge sample processing and examination.
2. Take the sludge sample from the cold box to the top of clean and white surface lab bench/table.
3. Open carefully the sample container and homogenize well using metal spatula.

**Note:** Inspect maggots escaping from the sludge sample and protect lab environment contamination.

4. Put graduated plastic cup on top of a digital weight scale, tape to zero and weigh 10 gm of the sludge sample using wooden/steel spatula.
5. Fill tap water to the mark of 80ml^(+)^ and mix well using wooden spatula (new).

**Note:** Pre-sieve (strain) the fecal sludge sample three times using tea strainer if it is sticky (easily undigestible) to withhold the large debris.

6. Prepare Fluke Catcher sieve set in their order of sieve size (#1=189 μm, #2= 104 μm and #3=59 μm) from top to bottom.

**Note:** Detach Fluke Catcher sieve #4=18 μm, and instead use 20 μm sieve (100 mm wide)

7. Hold the Fluke Catcher sieve set on top of graduated 3 litre plastic jug in a slight slanted position. **Note:** The top of the Fluke Catcher sieve set away from, and the holes to the personnel side.
8. Add the well mixed sludge sample from the graduated plastic cup to the top of the Fluke Catcher sieve set and shake it using the other hand and/or wall of the jug.

**Note:** Add the sludge sample gradually to prevent overflow.

9. Rinse the graduated plastic cup 3 to 5 times and repeat procedure **#8**.
10. Add tap water directly to the top of each sieve of the Fluke Catcher by removing sequentially (sieve #1 to #3) until visually pure water is passed through the sieves to the plastic jug.

11. Pour the collected samples in the graduated 3 litre plastic jug on 20μm sieve carefully (handling by a hand and shake with the other hand).
12. Rinse the jug 3 times and pour over the 20μm sieve (repeat procedure **#11**).
13. Collect samples (retentate) remaining on top of the 20μm sieve to one side and transfer to plastic cup by rinsing using tap water.
14. Transfer the collected sample (including the rinsed) from the plastic cup to 50ml plastic conical tube(s).
15. Centrifuge the sample in the conical tube at 9000 rpm for 5 minutes.
16. Decant the supernatant until the sediment remained will be ≤ 5ml.

**Note:** Re-centrifuge the sample if the sediment is > 5ml and decant the supernatant to ≤ 5ml.

17. Mix well the sediment and re-fill to 5ml if it is < 5ml.
18. Take 50μl of the sediment, put on top of microscope slide and cover with coverslip.

**Note:** Mix well the sediment between preparation of individual slide smear.

19. Examine the prepared smear and count eggs of STHs and *S. mansoni*.
