# Supplementary material for: Development, field-testing and optimization of tools to quantify soil-transmitted helminths in fecal sludge from school pit latrines in Ethiopia
Source: PLoS Negl Trop Dis. 2026 Sep 15;20(9):e0014724. doi: 10.1371/journal.pntd.0014724 (PMC13592699; doi:10.1371/journal.pntd.0014724)
Supplement: Supplementary file 2 [file pntd.0014724.s002.docx]

**S2 Text :** **Theoretical framework to decompose sources of variation in egg counts.**

Egg counts () in samples from pit latrines were modelled with a multi-level Poisson

regression model, accounting for variation between slides (index), microscopists (),

repeated sample preparation based on the same sludge sample (), pit latrine holes

within the same school (), schools (), species (), and an optional set of predictors (,

fixed effects):

$$y_{qijkms}\sim\text{Poisson}\left( \lambda_{qijkm} \right)$$

(Eq. 1)

$$\lambda_{qijkm}=\text{weight}_{\text{solids,}qijk}\cdot\mu_{qijk}\cdot M_{ijkm}$$

(Eq. 2)

$$M_{ijkm}\sim\Gamma\left( k_{M},k_{M} \right)$$

(Eq. 3)

$$\mu_{qijk}\sim\Gamma\left( k_{K},\frac{k_{K}}{\mu_{qij}} \right)$$

(Eq. 4)

$$\mu_{qij}\sim\Gamma\left( k_{J},\frac{k_{J}}{\mu_{qi}} \right)$$

(Eq. 5)

$$\log\left( \mu_{qi} \right)=X\beta$$

(Eq. 6)

Note that the unit of $\mu_{qi}$ through $\mu_{qijk}$ is eggs per gram solids, and that we use $\text{weight}_{\text{solids}}$ (unit: gram) as an offset to translate this to the expected number of counted eggs $\lambda_{qijkm}$ in a slide. Further note that the relative variation $M_{ijkm}$ caused by microscopists is assumed to be perfectly correlated across different species’ eggs counted within the same slide. That is, we assumed that $M_{ijkm}$ represents the attention or effort of a microscopists to count any eggs in that particular set of slides from a particular sample. And we assume that this attention or effort per microscopist is not correlated across different sets of slides.

To quantify the gamma-distributed random effects (Eqs. 3–5), the contribution of each variance component (microscopists $M$, sample processing $K$, and hole-to-hole variation $J$) towards the potential overdispersion in egg counts per species within a school was expressed in terms of the coefficient of variation per component (${CV}_{M}$, ${CV}_{K}$, ${CV}_{J}$). For a gamma distribution with shape parameter $k$, this is defined as $CV=k^{-1/2}$ (and conversely, $k={CV}^{-2}$). The total coefficient of variation over all three levels is then ${CV}_{total}=\sqrt{\prod_{c} \left( {CV}_{c}^{2}+1 \right)-1}$ ($c\in\left\{ M, K, J \right\}$). To avoid unreasonably thick tails on the push-forward prior for the total coefficient of variation ${CV}_{total}$, we formulated a prior for ${CV}_{total}$ and a prior for the relative contribution $\kappa_{i}$ of each individual variance component to ${CV}_{total}$, such that: ${CV}_{c}=\sqrt{\left( {CV}_{total}^{2}+1 \right)^{\kappa_{c}}-1}$. Here, we satisfy the required condition $\left( {CV}_{total}^{2}+1 \right)=\prod_{i} \left( {CV}_{total}^{2}+1 \right)^{\kappa_{c}}$ by defining $\kappa_{1:3}$ as a simplex vector (i.e., $\sum_{c} \kappa_{c}=1$, where ${0\leq\kappa}_{c}\leq1$). We note that the parameters ${CV}_{total}$ and $\kappa_{1:3}$ were between all three worm species present; as one worm species dominated the data, there were not enough egg counts to reliably estimate ${CV}_{total}$ and $\kappa_{1:3}$ per worm species. For ${CV}_{total}$, we defined a weakly informative half-normal prior distribution $N^{+}\left( 0,5 \right)$. For $\kappa_{1:3}$ we specified a Dirichlet prior with shape 1 for all elements (i.e., $Dir\left( \alpha_{1:3} \right)$, where $\alpha_{i}=1$), which is a uniform prior over the simplex parameter spaceFor the school- and species-specific average egg count, we defined a weakly informative lognormal prior $N^{+}\left( \mu_{qi},\sigma_{\mu} \right)$ with its mean set to the empirical mean of the data and the standard deviation set to 1.

Optionally, via the linear predictor $X\beta$, the model also accounted for sampling depth and pit latrine holes assigned to boys and girls, using improper uniform priors for $\beta$ on the logarithmic scale (i.e., $P_{prior}\left( \beta\right)\propto\frac{1}{\beta}$).

In a sensitivity analysis, we replaced all the gamma distributions for random effects (Eqs. 3–5) with log-normal random effects:

$$M_{ijkm}\sim lognormal\left( 0,\sigma_{M} \right)$$

(Eq. 3)

$$\mu_{qijk}\sim\mathrm{lognormal}\left( \log\left( \mu_{qij} \right),\sigma_{K} \right)$$

(Eq. 4)

$$\mu_{qij}\sim\mathrm{lognormal}\left( \log\left( \mu_{qi} \right),\sigma_{J} \right)$$

(Eq. 5)

Here, the standard deviation $\sigma_{c}$ of each of the random effects ($c\in\left\{ M, K, J \right\}$) is defined as a function of the corresponding ${CV}_{c}$ and $\kappa_{c}$, where $\sigma_{c}=\sqrt{\kappa_{c}\cdot\log\left( {CV}_{total}^{2}+1 \right)}$. In this version of the model, the estimated value of $\mu_{qi}$ represents the geometric mean egg count instead of the arithmetic mean.

**Fig A: Observed association between average and variance of Ascaris egg counts in a laboratory setting.** Each point represents the average and variance across four repeated slides based on the same 50 µL of processed fecal sludge that was spiked with a known quantity of eggs (symbols). The diagonal dashed line is the identity line and indicates the expected association between mean and variance if the repeated egg counts across slides follow a Poisson distribution.

**Fig B : Association between average and variance of egg counts across slides, processed samples, and pit latrine holes in field settings.** In the left panel (“Slides”), each point represents the average and variance of slide level egg counts across four repeated slides (each 50 µL) taken from the same processed 10 g of fecal sludge. In the middle panel (“Samples”), each point represents a single fecal sludge sample (500 to 1,300 mL) from which four subsamples of each 10 g were processed and examined with 4 slides per 10 g of fecal sludge. Note that this was only done in three out of the six schools. The average and variance for each point were calculated by summing up the egg counts of each set of 4 slides (representing 10 g of processed sludge) and then calculating the average and variance across the 4 sets of 10 g of processed fecal sludge. In the right panel (“Holes”), each point represents a school from which four squat holes were sampled from the same pit latrine; multiple points per school represent different sampling depths. For each hole, all egg counts across repeated sample preps and repeated slides were added up, except for Dilfire, where we only used the results from the first sample processing as quadruple sample processing was only done for two of the four holes. As such, each point represents the average and variance of egg counts across holes within the same school. Diagonal dashed lines indicate the expected association between mean and variance if egg counts would follow a Poisson distribution; points above that line indicate overdispersion of egg counts.

**Fig C: Observed association between average and variance of egg counts from superficial and deep samples from pit latrine holes in field settings.** Each point represents a school from which four pit latrine holes were sampled; multiple points per school represent different sampling depths. For each hole and sampling depth, all egg counts across repeated sample processing and repeated slides were added up, except for Dilfire, where we only used the results from the first sample processing as quadruple processing was only done for two of the four holes. As such, each point represents the average and variance of egg counts across holes within the same school. The diagonal dashed line is the identity line and indicates the expected association between mean and variance if hole-level total egg counts would follow a Poisson distribution. Note that for the school in Hirmata, the sludge type varied between holes (semi-solid or slurry, see [THE PREVIOUS SUPPLEMENTAL FIGURE]), whereas for the school in Jimma, all sludge samples were of the semi-solid type.

**Fig D : Observed association between average and variance of egg counts across semi-solid and slurry samples in field settings.** Each point represents a single sludge sample (500 to 1,300 mL) from which four subsamples of 10 gr each were processed and examined with 4 slides per 10 gr of sludge. Note that this was only done in three out of the six schools. The average and variance for each point were calculated by summing up the egg counts of each set of 4 slides (representing 10 gr of processed sludge), and then calculating the average and variance across the 4 repeated sets of 10 gr of processed sludge. The diagonal dashed line is the identity line and indicates the expected association between mean and variance if the repeated egg counts across processed samples of 10 gr of sludge would follow a Poisson distribution.

**Table A. The analytical performance of our modified diagnostic method to detect Ascaris eggs in fecal sludge samples**

| N of eggs spiked | Sludge type | N of replicates | N of slides per replicate | N of replicates with a positive test result | Percent replicates with a positive test result  (95% CI)* | Median percent eggs recovered (min, max) | Range in percent eggs recovered |
| --- | --- | --- | --- | --- | --- | --- | --- |
| 50 | Liquid | 8 | 4 | 6 | 75 (40.9-92.9) | 50 (0, 200) | 200 |
|  | Semi-solid | 8 | 4 | 4 | 50 (21.5-78.5) | 25 (0, 100) | 100 |
|  | Slurry | 8 | 4 | 7 | 87.5 (52.9-97.8) | 50 (0, 150) | 150 |
| 100 | Liquid | 8 | 4 | 7 | 87.5 (52.9-97.8) | 75 (0, 125) | 125 |
|  | Semi-solid | 8 | 4 | 8 | 100 (67.6-100) | 75 (25, 100) | 75 |
|  | Slurry | 8 | 4 | 7 | 87.5 (52.9-97.8) | 62 (0, 150) | 150 |
| 200 | Liquid | 8 | 4 | 8 | 100 (67.6-100) | 100 (38, 175) | 137 |
|  | Semi-solid | 8 | 4 | 8 | 100 (67.6-100) | 50 (12, 100) | 88 |
|  | Slurry | 8 | 4 | 8 | 100 (67.6-100) | 50 (25, 150) | 125 |
| 400 | Liquid | 8 | 4 | 8 | 100 (67.6-100) | 81 (50, 100) | 50 |
|  | Semi-solid | 8 | 4 | 8 | 100 (67.6-100) | 50 (19, 69) | 50 |
|  | Slurry | 8 | 4 | 8 | 100 (67.6-100) | 59 (44, 75) | 31 |

*****95%CI is calculated using a binomial exact test

**Table B. The prevalence and concentration of Ascaris, Trichuris and S. mansoni in the fecal sludge across six schools**

|  | | **N** | ***Ascaris*** | |  | ***Trichuris*** | |  | ***S. mansoni*** | |
| --- | --- | --- | --- | --- | --- | --- | --- | --- | --- | --- |
|  |  |  | Prevalence (% ; 95%CI) | Median concentration (EPG_TS ;_ min ; max) |  | Prevalence (% ; 95%CI) | Median concentration (EPG_TS ;_ min ; max) |  | Prevalence (% ; 95%CI) | Median concentration (EPG_TS ;_ min ; max) |
| **School** | |  |  |  |  |  |  |  |  |  |
|  | Dilfire | 32 | 100  (89.3; 100) | 2765  (870; 42,890) |  | 25.0  (13.3; 42.1) | 0  (0; 155) |  | 50.0  (33.6; 66.4) | 7  (0; 53) |
|  | Ginjo | 16 | 100  (80.6; 100) | 4,220  (3,000; 5,606) |  | 100  (80.6; 100) | 14  (13; 30) |  | 75.0  (50.5; 89.8) | 20  (0; 62) |
|  | Hirmata | 32 | 100  (89.3; 100) | 7,244  (2,556; 16,722) |  | 100  (89.3; 100) | 210  (28 ; 694) |  | 0  (0; 10.7) | 0 |
|  | Jimma | 16 | 100  (80.6; 100) | 6,278  (4,668; 12,747) |  | 100  (80.6; 100) | 73  (48; 148) |  | 0  (0; 19.4) | 0 |
|  | Kito | 32 | 100  (89.3; 100) | 2,614  (250; 9,286 |  | 12.5  (5.0 ; 28.1) | 0  (0; 227) |  | 0  (0; 10.7) | 0 |
|  | Mendera | 32 | 100  (89.3; 100) | 4,821  (1,789; 7,458) |  | 100  (89.3; 100) | 97  (39; 174) |  | 100  (89.3; 100) | 144  (53; 226) |
| **Usage** | |  |  |  |  |  |  |  |  |  |
|  | Boys | 80 | 100  (95.4; 100) | 3,949  (250; 12,747) |  | 65.0  (54.1; 74.5) | 38  (0; 174) |  | 40.0  (30.0; 51.0) | 0  (0; 184) |
|  | Girls | 80 | 100  (95.4; 100) | 4,527  (278, 16,722- |  | 70  (59.2; 78.9) | 50  (0; 694) |  | 35.0  (25.5; 45.9) | 0  (0; 226) |
| **Depth** | |  |  |  |  |  |  |  |  |  |
|  | Deep | 64 | 100  (94.3; 100) | 5,563  (870: 11,826) |  | 56.2  (44.1; 67.7) | 59  (0; 500) |  | 31.2  (21.2; 43.4) | 0  (0; 226 |
|  | Superficial | 96 | 100  (96.2; 100) | 41,023  (250; 16722) |  | 75.0  (65.5; 82.6) | 40  (0; 694) |  | 41.7  (32.3; 51.7) | 0  (0; 184) |

**Table C. Historical data on prevalence and infection intensity for Ascaris and Trichuris**. This table summarizes the historical (2015 aen 2018) prevalence and infection intensity (measured as eggs per gram of stool (EPG)) for the six schools that were include in the present study and the corresponding eggs per gram of fecal sludge total solids (EPG_TS_) observed in 2023.

| **School** | ***A. lumbricoides*** | | | | |  | ***T. trichiura*** | | | |  |
| --- | --- | --- | --- | --- | --- | --- | --- | --- | --- | --- | --- |
|  | **2015** | | **2018** | | **2023** |  | **2015** | | **2018** | | **2023** |
|  | **Prev (%)** | **EPG** | **Prev (%)** | **EPG** | **EPG_TS_** |  | **Prev (%)** | **EPG** | **Prev (%)** | **EPG** | **EPG_TS_** |
| Dilfire | 28.3 | 2,168.0 | 11.7 | 290.0 | 1,810.7 |  | 40.0 | 70.0 | 20.0 | 17.6 | 23.65 |
| Ginjo | 33.3 | 2,411.2 | 21.7 | 2,483.2 | 4,261.7 |  | 35.0 | 200.4 | 20.0 | 26.8 | 17.48 |
| Hermata | 26.7 | 2,679.2 | 10 | 1,236.8 | 9,419.0 |  | 40.0 | 200.4 | 23.3 | 32.4 | 265.86 |
| Jimma | 35.0 | 2,345.2 | 28.3 | 4,550.8 | 8,098.3 |  | 45.0 | 149.2 | 38.3 | 162.8 | 99.24 |
| Kitto | 25.0 | 6,929.2 | 18.3 | 1,389.6 | 3,185.3 |  | 36.7 | 174.8 | 16.7 | 77.2 | 28.41 |
| Mendera | 36.7 | 3,274.0 | 6.7 | 3,509.6 | 4,929.8 |  | 41.7 | 206.4 | 11.7 | 50.4 | 105.19 |

**Fig E: Associations between historical Ascaris and Trichuris infection data from school-based surveys (2015 and 2018) and parasite egg concentrations measured in fecal sludge in 2023.** Historical data include prevalence (%) and infection intensity (eggs per gram, EPG) from the six schools included in the study. Fecal sludge infection intensity is expressed as eggs per gram of total solids (EPG_TS_). Panels **A** and **B** relate stool EPG to fecal sludge EPG_TS_ for Ascaris and Trichuris, respectively, while panels **C** and **D** relate historical prevalence to fecal sludge EPG_TS_ for the respective parasites. Each point represents a study school, and colors denote the historical survey year (2015 or 2018).
